# Correction: Lee et al. Prioritization of Critical Factors for Surveillance of the Dissemination of Antibiotic Resistance in *Pseudomonas aeruginosa*: A Systematic Review. *Int. J. Mol. Sci.* 2023, *24*, 15209

**DOI:** 10.3390/ijms26094166

**Published:** 2025-04-28

**Authors:** Jung Hun Lee, Nam-Hoon Kim, Kyung-Min Jang, Hyeonku Jin, Kyoungmin Shin, Byeong Chul Jeong, Dae-Wi Kim, Sang Hee Lee

**Affiliations:** 1 National Leading Research Laboratory of Drug Resistance Proteomics, Department of Biological Sciences, Myongji University, Yongin 17058, Republic of Korea; junghunlee@mju.ac.kr (J.H.L.); cardgame79@gmail.com (K.-M.J.); ehdqhdl1@naver.com (H.J.); hiu980305@naver.com (K.S.); bcjeong@mju.ac.kr (B.C.J.); 2 Division of Life Sciences, Jeonbuk National University, Jeonju 54896, Republic of Korea; krkimdoller@jbnu.ac.kr

The journal’s Editorial Office and Editorial Board are jointly issuing a resolution and removal of the *Journal Notice* linked to this article [1], as well as an update to the original publication. Following concerns raised about the integrity of the peer review, the Editorial Office has conducted a post-publication peer review of this article. This process included the recruitment of a new independent reviewer and was supervised by the original Academic Editor to ensure full compliance with MDPI’s Editorial Process (https://www.mdpi.com/editorial_process (accessed onaccessed on 18 March 2025)).

As a result of this process, the Academic Editor and the authors have agreed to update the following aspect(s) of this publication:

Reviewer report 1 was removed from the peer review record and a new report was uploaded to the peer review record (https://www.mdpi.com/1422-0067/24/20/15209/review_report (accessed on 18 March 2025)).

Based on the new review report, the following corrections have been made to the original publication:

A text correction:

Methods Section, 2.2. Genome Database Survey, Paragraph 4 to add the detailed methods for the genome database survey;
3. Results, 3.2.4. Mobile ARGs, Paragraph 2 to correct the typos;
4. Discussion, Paragraph 2 to explain the significance of mobile ARGs defined in this study;
References to add two missing references [2,3].

With this update, the Academic Editor is satisfied that the Editorial Process relating to this article has been completed as per MDPI’s Editorial Process policy. The Editorial Office would like to thank the authors for their collaboration during this process.
