# Supplementary material for: Prioritization of Critical Factors for Surveillance of the Dissemination of Antibiotic Resistance in Pseudomonas aeruginosa: A Systematic Review
Source: Int J Mol Sci. 2023 Oct 15;24(20):15209. doi: 10.3390/ijms242015209 (PMC10607276; doi:10.3390/ijms242015209)
Supplement: Supplementary file 1 [file ijms-24-15209-s001.zip › Supplementary Tables S1-S4, S8.pdf]

Table S1. Characterization of mobile genetic elements (MGEs), integrons, associated with antibiotic resistance genes (ARGs) in *P. aeruginosa*

Source	MGEs	ARGs associated	Geographic region	Year	Reference
Human	class 1 integron	*aacA4, aadA7, bla*$_{OXA-56}$	Brazil	2017	[52]
Environment	class 1 integron	*aadA7, bla*$_{TEM-116}$, *sul1*	Croatia		[53]
Human	class 1 integron	*bla*$_{VIM-2}$, *qacF*, *aacA4*, *catB3*, *bla*$_{OXA-30}$, *aadAI*, *qacEΔ1*	Korea		[54]
Human	class 1 integron	*bla*$_{IMP-10}$, *aacA7*	China	2012	[55]
Human	class 1 integron	*aac(6')-33*, *bla*$_{GES-19}$, *aac(6')-lb-cr*, *bla*$_{OXA-2}$, *qac*, *aadA1*, *qacEΔ1*, *sul1*	Mexico	2017	[56]
Human	class 1 integron	*bla*$_{GES-1}$, *acc(6')-Ib*, *aph(3')-XV*, *sul1*, *floR*, *tetR*, *tet*(G)	China	2010	[57]
Human	class 1 integron	*aadB*	Thailand	2007, 2008	[58]
	class 1 integron	*aacA7*, *aacA7b*			
	class 1 integron	*aac(3)-Ic-smr*, *cmlA5b*			
	class 1 integron	*aadA6*			
	class 1 integron	*aacA7*, *aacA7*			
	class 1 integron	*bla*$_{PSE-1}$, *aadA2*			
	class 1 integron	*aadB*, *cmlA6*, *aadA15*			
	class 1 integron	*blaVEB-2*, *aadB*, *arr*, *cmlA5*, *bla*$_{OXA-10}$, *aadA1*			
	class 1 integron	*sul1*, *ant(2'')-Ia*, *aadA6*, *aac(6')-Ib'*, *aadA2*, *bla*$_{OXA}$, *bla*$_{CARB}$, *bla*$_{OXA}$			[59]
Human	class 1 integron	*bla*$_{GES-5}$, *bla*$_{GES-5}$, *bla*$_{GES-5}$, *bla*$_{GES-5}$	China	2012	[60]
Human	class 1 integron	*aac(6')-33*, *bla*$_{GES-19}$, *aacA4*, *bla*$_{OXA-2}$, *aadA1*, *qacEΔ1*, *sul1*	Brazil	2016	[61]
Human	class 1 integron	*aadB*, *aac6-II*, *bla*$_{PSE-1}$	China	2006, 2007	[62]
	class 1 integron	*dfrA17*, *aadA5*			
	class 1 integron	*aac6-II*			
Human	class 1 integron	*aadA7*, *qacEΔ1*, *sul1*	Cyprus		[63]
Salmon	class 1 integron	*aac(6')-31*, *qacH*, *bla*$_{OXA-2}$	Chile	2008-2010	[64]
Human	class 1 integron	*qacE*, *dfrAS1*, *aadA1*, *bla*$_{OXA-10}$, *cmlA1*, *aac(6')-Ib-cr*, *bla*$_{SIM-1}$	United Kingdom	2019	[65]
	class 1 integron	*dfrA1*, *aadA1*, *bla*$_{OXA-10}$, *aac(6')-Ib-cr*, *bla*$_{SIM-1}$			

Human	class 1 integron	*aadB*, *cmlA6*, $bla_{\text{OXA-4}}$, *aadA2*	Japan	2014, 2018	[66]
	class 1 integron	$bla_{\text{OXA-4}}$, *aadA2*, *aadB*			
	class 1 integron	$bla_{\text{OXA-4}}$, *aadA2*			
	class 1 integron	*aacA31*			
Human	class 1 integron	*aacA4*	Czech	2007	[67]
	class 1 integron	*aadB*, *aadA13*			
	class 1 integron	*aadB*			
	class 1 integron	*aacA7*, *aadA6*			
	class 1 integron	*aacA7*, *aacA7*, *aadA6*			
	class 1 integron	*aacA7*, *aadA6*			
	class 1 integron	*aacA7*			
	class 1 integron	*aacA8*, $bla_{\text{OXA-2}}$, *aacA7*			
	class 1 integron	*aacA8*, $bla_{\text{OXA-2}}$, *aacA7*, *aacA7*, *aadA6*			
	class 1 integron	*aacA4*, *catB10*			
	class 1 integron	*aacA4*, $bla_{\text{PSE-1}}$, *aadA2*			
	class 1 integron	*aacA4*, *aacA7*, *aacA7*			
	class 1 integron	*aacA4*, $bla_{\text{IMP-7}}$, *aacA4*, *aacA4*, $bla_{\text{OXA-2}}$			
	class 1 integron	*aadB*, *cmlA*			
	class 1 integron	$bla_{\text{OXA-31}}$, *aadA2*, *cmlA*			
Human	class 1 integron	$bla_{\text{OXA-101}}$, *aacA4*, *catB*	Colombia	2005, 2008	[68]
Dog	class 1 integron	*aadA*	U.S.A	2003, 2006	[69]
Human	class 1 integron	*sul1*, *emrE*, *aadA*, $bla_{\text{OXA-10}}$, *cmlA5*, *arr-2*, *ant(2')-Ia*, $bla_{\text{VEB-1}}$	Thailand		[70]
Human	integron	$bla_{\text{VIM-2}}$	Costa Rica	2010	[71]
	integron	$bla_{\text{IMP-18}}$			
Human	class 1 integron	*aadB*	Iran	2015, 2016	[72]
	class 1 integron	*aadA6*			
	class 1 integron	*aadB*, *aadA1*, $bla_{\text{OXA-10}}$, *aac(6)-II*, $bla_{\text{OXA-10}}$			
	class 1 integron	*aac(6)-II*, *aacA4*, $bla_{\text{OXA-10}}$, $bla_{\text{VIM-6}}$, *aac(6)-Ib*			

	class 1 integron	*aacA4*, *catB10*			
	class 1 integron	*aacA4*, *bla*$_{OXA-10}$			
	class 2 integron	*dfrA1*			
	class 3 integron	*aacA7*, *aacA4*, *bla*$_{OXA-2}$			
Human	class 1 integron	*bla*$_{OXA-10}$, *aadB*, *bla*$_{VIM-2}$, *aadB*, *bla*$_{OXA-10}$	Tunisia		[73]
Human	class 1 integron	*bla*$_{GES-13}$, *bla*$_{VIM-2}$, *bla*$_{VIM-6}$, *bla*$_{OXA-10}$, *aacA(6')-Ib, aacA(6')-II, aadA6, gcuD*	Malaysia	2014	[74]
	class 1 integron	*bla*$_{GES-20}$, *bla*$_{IMP-4}$, *bla*$_{VIM-2}$, *bla*$_{VIM-11}$			
Human	class 1 integron	*bla*$_{GES-2}$	Korea	2016, 2017	[75]
Human	class 1 integron	*aadA22*, *aadB*	China	2018	[76]
	class 1 integron	*aac(6')-II*, *bla*$_{PSE-1}$			
	class 1 integorn	*dhfr2*, *aacA4*, *aadA1*			
	class 1 integron	*aacA4*, *bla*$_{OXA-101}$, *aadA5*			
	class 1 integron	*qnrVC*, *gcu165*, *arr2*, *dfrA22e*, *aacA4*, *gcu35*, *bla*$_{OXA-1}$, *catB3*			
	class 1 integron	*aacA4*, *bla*$_{OXA-101}$, *aadA5*			
Human	class 1 integron	*qacEΔ1*, *aadB*, *aadA10e*, *sul1*, *bla*$_{OXA-10}$	U.S.A	1999-2018	[77]
Human	class 1 integron	*bla*$_{VIM-2}$, *bla*$_{PIB-1}$, *aacA4*	Greece	2005	[78]
Human	class 1 integron	*bla*$_{VIM-2}$, *aacA7*, *aacC1*, *aacA4*, *qacEΔ1*, *sul1*	Portugal	2008, 2010	[79]
Human	class 1 integron	*bla*$_{IMP-1}$	China	2016	[80]
	class 1 integron	*bla*$_{IMP-8}$			
	class 1 integron	*bla*$_{CTX-M-14}$			
Human	class 1 integron	*bla*$_{VIM-5}$	Bangladesh	2016	[81]
	class 1 integron	*aacA8*, *bla*$_{OXA-2}$, *aacA7*			
Human	class 1 integron	*bla*$_{GES-2}$, *aacA4*, *gcuE15*, *aphA15*	Australia	2006, 2007	[82]
Human	class 1 integron	*bla*$_{IMP-7}$, *aacA7*, *bla*$_{VIM-2}$	Malaysia	2002-2008	[83]
	class 1 integron	*bla*$_{IMP-4}$, *aadA6*			
Human	class 1 integron	*aadB*	Spain	2005-2008	[84]
Human	class 1 integron	*aacA4*, *aadA1*	China	2009, 2010	[85]

	class 1 integron	*bla*OXA-31, *aadA2*			
	class 1 integron	*aadA1*, *arr*, *catB3*			
	class 1 integron	*cmlA5*, *cmlA*, *aadA1*			
Dog	class 1 integron	*aacA4*	Brazil	2010-2012	[86]
	class 1 integron	*aadA6*			
Dog	class 1 integron	*aacA7*, *bla*VIM-2, *dfrB5*, *aacC5b*	Korea	2013-2017	[87]
	class 1 integron	*dfra*			
Human	class 1 integron	*aac(6')-Iaf*, *bla*IMP-1, *qacEΔ1*, *sul1*	Japan	2007	[88]
Human	class 1 integron	*qacEΔ1*, *sul1*, *cmlA9*, *qacEΔ1*, *tetR*, *tetA*(G), *sul1*	U.S.A	2007	[89]
Human	class 1 integron	*bla*VIM-4	Greece	2001	[90]
Human, Environment	class 1 integron	*aph(6)-Id*, *aph(3'')-Ib*, floR, *sul2*	Singapore	2019, 2020	[91]
	class 1 integron	*bla*NDM-1, *msr*(E), *floR*			
	class 1 integron	*aac(3)-Id*, *aac(6')-Il*, *aadA6*, *aadA11*, *dfrB5*, *qnrVC1*			
Human	class 1 integron	*bla*OXA-10, *aacA4*, *bla*VIM-2, *smr-2*, *qacEΔ1*	Greece	2011	[92]
	class 1 integron	*bla*VIM-2, *aacA7*, *dfr*, *qacEΔ1*			
Human	class 1 integron	*bla*IMP-1, *aacA28*, *aadA1*	Japan	2004, 2006	[93]
	class 1 integron	*bla*IMP-1			
	class 1 integron	*aacA7*, *fosI*, *bla*IMP-41, *qacG*			
	class 1 integron	*aacA7*, *bla*IMP-11, *bla*OXA-2, *qacG*			
	class 1 integron	*bla*IMP-6, *fosE*			
	class 1 integron	*fosE*, *aacA31*, *bla*VIM-2			
	class 1 integron	*bla*IMP-10, *aadA2*, *bla*OXA-47			
	class 1 integron	*bla*IMP-10, *aacA1*, *gcuG*			
	class 1 integron	*bla*IMP-1, *aacA4*, *aacA4*, *catB6*, *blaCARB-12*			
	class 1 integron	*bla*IMP-1, *aacA4*, *aacA1*, *gcuG*, *aadA1a*			
public	class 1 integron	*bla*IMP-1, *aadB*	public	public	[94]
	class 1 integron	*bla*IMP-1, *aacA4*			

Human	class 1 integron	*gar*, *qacEΔ1*	Italy		[95]
Human	class 1 integron	*aacA4*, *bla*$_{\text{OXA-28}}$	France	2004	[96]
	class 1 integron	*aacA4*, *gcuF1*, *bla*$_{\text{OXA-28}}$			
Human	class 1 integron	*bla*$_{\text{IMP-18}}$, *aadA1*, *bla*$_{\text{OXA-2}}$, *qacEΔ1*, *sul1*	Mexico	2013-2015	[97]
	class 1 integron	*bla*$_{\text{IMP-56}}$, *aadA1*, *bla*$_{\text{OXA-2}}$, *qacEΔ1*, *sul1*			
	class 1 integron	*aacA7*, *bla*$_{\text{IMP-62}}$, *qacH*, *aacA4*, *aacA1*, *bla*$_{\text{OXA-2}}$			
Human	class 1 integron	*qacEΔ1*, *sul1*	Iran	2016-2018	[98]
	class 1 integron	*aadB*, *qacEΔ1*, *sul1*			
	class 1 integron	*aadA6*, *qacEΔ1*, *sul1*			
	class 1 integron	*aacA4*, *catB*, *qacEΔ1*, *sul1*			
	class 1 integron	*aacA4*, *bla*$_{\text{OXA-10}}$, *qacEΔ1*, *sul1*			
	class 1 integron	*bla*$_{\text{OXA-31}}$, *aadA2*, *qacEΔ1*, *sul1*			
	class 1 integron	*bla*$_{\text{OXA-10}}$, *aacA4*, *bla*$_{\text{VIM-1}}$, *qacEΔ1*, *sul1*			
	class 1 integron	*aac(3)-Ic*, *aacA5*, *cmlA5*, *qacEΔ1*, *sul1*			
	class 1 integron	*aacA5*, *aadA1*, *cmlA5*, *qacEΔ1*, *sul1*			
Human	class 1 integron	*aacA4*, *aphA15*, *aadA1*, *qacEΔ1*, *sul1*	Portugal	2004	[99]
Human	class 1 integron	*bla*$_{\text{VIM-2}}$, *aac(6')-II*, $_{\text{dfrB-5}}$, *aac(3')-Id*	Saudi Arabia	2018	[100]
Human	class 1 integron	*aac(6')-Ib-7*, *bla*$_{\text{IMP-45}}$, *bla*$_{\text{OXA-1}}$, *catB3*, *qacEΔ1*, *sul1*	China	2015-2017	[101]
	class 1 integron	*qnrVC1*, *arr-2*, *dfrA22*, *qacEΔ1*, *sul1*			
Human	Partial class 1 integron	*bla*$_{\text{IMP-4}}$, *bla*$_{\text{DIM-1}}$, *bla*$_{\text{OXA-1}}$, *bla*$_{\text{GES-2}}$, *bla*$_{\text{VIM-2}}$	Mexico	2023	[46]
	Class 1 integorn	*bla*$_{\text{OXA-101}}$, *bla*$_{\text{CTX-M-30}}$, *bla*$_{\text{TEM-1b}}$			
Human	class 1 integron	*bla*$_{\text{IMP-56}}$, *aadA1*, *bla*$_{\text{OXA-2}}$	Mexico	2023	[102]
Snake	class 1 integron	*tetA*(C), *tetA*(R), *qacH*, *aadB*, *cmlA10*, *aadA2*, *qacEΔ1*, *sul1*	France	2004	[103]
Human	class 1 integron	*bla*$_{\text{OXA-10}}$, *aac(6')-Ib*, *bla*$_{\text{IMP-8}}$, *aac(6')-Ib*, *aph(3')-XV*, *aadA10*, *bla*$_{\text{OXA-2}}$, *sul1*	Germany	2009	[104]
Human, Environment	class 1 integron	*bla*$_{\text{IMP-19}}$, *aac(6')-Ib*, *aadA13*, *qacEΔ1*, *sul1*	France	2009, 2013, 2015, 2016	[105]
	class 1 integron	*bla*$_{\text{IMP-19}}$, *aac(6')-Ib*, *aadB*, *aadA13*, *qacEΔ1*, *sul1*			
	class 1 integron	*bla*$_{\text{IMP-19}}$, *qacEΔ1*, *sul1*			

	class 1 integron	*aac(6')-Ib*, *bla*$_{IMP-19}$, *aac(6')-Ib*, *qacEΔ1*, *sul1*			
	class 1 integron	*aac(6')-Ib*, *qacG*, *aac(6')-Ib*, *bla*$_{IMP-19}$, *qacEΔ1*, *sul1*			
	class 1 integron	*aac(6')-Ib*, *bla*$_{IMP-19}$, *qacEΔ1*, *sul1*			
	class 1 integron	*bla*$_{IMP-19}$, *aac(6')-Ib*			
Human	class 1 integron	*aadB*, *aadA13*	Hungary	2005-2007	[106]
	class 1 integron	*aadB*			
Human	class 1 integron	*bla*$_{IMP-18}$, *aadA1*, *bla*$_{OXA-2}$, *aadA1*, *qacEΔ1*	Mexico	2014	[107]
Human	class 1 integron	*aadA7*	Algeria	2014-2015	[108]
Human, Environment	class 1 integron	*aac(6')-Ib*, *bla*$_{PSE-1}$, *ant(3')-Ia*	Hungary	2008-2010	[109]
Human	class 1 integron	*bla*$_{VIM-7}$, *aacA4*, *bla*$_{OXA-46}$, *qacEΔ1*, *sul1*	Brazil	2013	[110]
Human	class 1 integron	*bla*$_{VIM-3}$, *aacA4*	Taiwan	2002-2006	[111]
	class 1 integron	*bla*$_{VIM-3}$, *aacA4*, *aadB*, *aacA4*			
Human, Environment	class 1 integron	*aac(6')-II*, *bla*$_{PIB-1}$, *aadA2*, *qacEΔ1*, *sul1*	Spain		[112]
Human	class 1 integron	*bla*$_{VIM-2}$, *sul1*	Russia	2012-2017	[113]
	class 1 integron	*aacA7*, *bla*$_{VIM-2}$, *dfrB5*, *aacC-A5*			
	class 1 integron	*aacA7*, *bla*$_{VIM-2}$, *dfrB5*, *aacC-A5*			
	class 1 integron	*aacA29a*, *bla*$_{VIM-2}$, *aacA29a*, *qacEΔ1*, *sul1*			
	class 1 integron	*aacA29a*, *bla*$_{VIM-2}$, *aacA29b*, *qacEΔ1*, *sul1*			
	class 1 integron	*aacA4*, *bla*$_{VIM-2}$, *qacEΔ1*, *sul1*			
Human	class 1 integron	*aadB*, *qacEΔ1*, *sul1*	Spain	2008-2010	[114]
	class 1 integron	*aadA7*, *qacEΔ1*, *sul1*			
	class 1 integron	*aadA6*, *qacEΔ1*, *sul1*			
	class 1 integron	*aac(3)-Ia*, *aadA1*, *qacEΔ1*, *sul1*			
	class 1 integron	*aac(3)-Ia*, *aadA1*, *qacEΔ1*, *sul1*			
	class 1 integron	*aac(6')-Ib*, *bla*$_{OXA-46}$, *qacEΔ1*, *sul1*			
	class 1 integron	*aac(6')-Ib*, *qacEΔ1*, *sul1*			
	class 1 integron	*aac(6')-Ib*, *bla*$_{VIM-2}$, *qacEΔ1*, *sul1*			

	class 1 integron	*bla*$_{VIM-2}$, *qacEΔ1*, *sul1*			
	class 1 integron	*bla*$_{VIM-2}$, *aac(6')-Ib*, *aadA1*			
	class 1 integron	*bla*$_{VIM-2}$, *aac(6')-Ib*, *aadA1*, *bla*$_{VIM-2}$, *qacEΔ1*, *sul1*			
Human	class 1 integron	*bla*$_{GES-5}$, *aadB*, *ahp(3')-VIa*, *bla*$_{NDM-1}$, *sul1*	Bulgaria	2017	[115]
Human	class 1 integron	*aacA55*, *aadA1*, *aacA55*, *aadA1a*, *aacA28*, *aadA1a*, *qacEΔ1*, *sul1*	India	2015	[116]
Human	class 1 integron	*aac(6')-Ib*, *bla*$_{PSE-1}$, *aadA2*, *qacEΔ1*, *sul1*	Portugal	2003, 2005	[117]
Human	class 1 integron	*bla*$_{VEB-1-like}$, *aadB*, *arr-2*, *cmlA5*, *bla*$_{OXA-10}$, *aadA1*, *qacEΔ1*	Thailand	1999	[118]
	class 1 integron	*bla*$_{VEB-1-like}$, *aadB*, *qacEΔ1*			
Human	class 1 integron	*bla*$_{VIM-4}$	Algeria	2016	[119]
Human	class 1 integron	*bla*$_{VIM-1}$, *aacA4*, *bla*$_{VIM-1}$, *bla*$_{VIM-1}$, *aadA1*, *qacEΔ1*, *sul1*	Spain	2006-2007	[120]
Human	class 1 integron	*aadB*	France	2011-2013	[121]
	class 1 integron	*aadA6*			
	class 1 integron	*aadB*, *aadA11*			
	class 1 integron	*dfrB1*			
	class 1 integron	*aacA4*, *aacC1d*, *gcuE*			
	class 1 integron	*aacA5*, *bla*$_{VIM-2}$			
	class 1 integron	*aacA4*, *cmlA1*, *bla*$_{VIM-2}$			
Human	class 1 integron	*aadA6*	Mexico	2004-2005	[122]
	class 1 integron	*aacA4*			
	class 1 integron	*aacA4*, *aadA1*			
	class 1 integron	*qacF*			
Human	class 1 integron	*aadB*, *aadA*, *qacEΔ1*, *sul1*	France	2001	[123]
Human	class 1 integron	*aacA4*, *bla*$_{PSE-1}$, *aadA2*	Italy	2000	[124]
Human	class 1 integron	*bla*$_{IMP-15}$,	Mexico	2004-2005	[125]
	class 1 integron	*bla*$_{VIM-2}$			
Human	class 1 integron	*aac(6')-Ib'*	Germany	2004	[126]
	class 1 integron	*aadB*, *aadA1*			

	class 1 integron	*aacA8*, *bla*$_{OXA-2}$, *aacA7*			
	class 1 integron	*aac(6')-Ib*, *bla*$_{PSE-1}$, *aadA2*			
	class 1 integron	*aac(6')-Ib'*, *aadA2*			
Human	class 1 integron	*bla*$_{NDM-1}$, *qnrVC1*, *aadA6*, *qacEΔ1*, *sul1*	Ghana	2015	[127]
Human	class 1 integron	*aacA4*, *bla*$_{IMP-5}$, *aacA4*	China	2000	[128]
Human	class 1 integron	*aacA7*, *bla*$_{OXA-198}$, *cmlA1*, *qacEΔ1*, *sul1*	Belgium	2010	[129]
Human	class 1 integron	*aac(6')-32*, *bla*$_{VIM-2}$, *qacEΔ1*	Spain	2003	[130]
Human	class 1 integron	*bla*$_{VIM-18}$, *qacEΔ1*, *sul1*	India		[131]
Human	class 1 integron	*aac(6')-Iaj*	Japan	2011	[132]
Human	class 1 integron	*dfrA1*, *bla*$_{VEB-1}$, *qacE*, *sul1*	India	2012	[133]
	class 1 integron	*dfrA12*, *bla*$_{VEB-1}$, *aad2*, *qacE*, *sul1*			
	class 1 integron	*bla*$_{VEB-1}$, *aaC-Ib*, *qacE*, *sul1*			
	class 1 integron	*dfrA17*, *bla*$_{VEB-1}$, *aadA5*, *qacE*, *sul1*			
Human	class 1 integron	*bla*$_{OXA-198}$, *catB7*	Belgium	2010-2013	[134]
Human	class 1 integron	*bla*$_{IMP-18}$, *aacA43*, *bla*$_{OXA-2}$, *gcuD*, *qacEΔ1*	Puerto Rico		[135]
	class 1 integron	*bla*$_{IMP-18}$, *aadA1b*, *bla*$_{OXA-224}$, *qacEΔ1*			
Human	class 1 integron	*aacA7*, *bla*$_{VIM-2}$, *aacC1*, *aacA4*, *qacEΔ1*, *sul1*	France	1998, 1997	[136]
	class 1 integron	*aacA29a*, *bla*$_{VIM-2}$, *aacA29b*, *qacEΔ1*, *sul1*			
Human	class 1 integron	*bla*$_{IMP-16}$, *aac(6')-30*, *aac(6')-Ib*, *aadAA1*, *qacEΔ1*	Latin America	1997-	[137]
Human	class 1 integron	*bla*$_{VIM-2}$, *aacA4*, *bla*$_{PIB-1}$, *aadA2*, *qacEΔ1*, *sul1*	Portugal	2000	[138]
Human	class 1 integron	*bla*$_{IMP-1}$, *aac(6')-Iae*, *aadA1*, *qacEΔ1*, *sul1*	Japan		[139]
Human	class 1 integron	*aacA29a*, *bla*$_{VIM-2}$	Colombia	2004	[140]
Human	class 1 integron	*bla*$_{VEB-1}$	Bangladesh, France	2003-2004	[141]
	class 1 integron	*aadB*, *dfrA1*			
Human	class 1 integron	*bla*$_{IMP-15}$, *aacA4*, *qacEΔ1*, *sul1*	Italy	2002, 2003	[142]
Human	class 1 integron	*aacA27*, *bla*$_{OXA-2}$, *qacEΔ1*, *sul1*	U.S.A	2010-2012	[143]
Human	class 1 integron	*aadB*, *bla*$_{OXA-205}$	Lithuania		[144]

Human	class 1 integron	*bla*$_{VIM-2}$, *aadB*, *dfrA1*, *qacE*, *sul1*	India	2012-2013	[145]
Human	class 1 integron	*aadB*, *aacA7*, *bla*$_{VIM-2}$, *dfrA1*, *qacE*, *sul1*	India	2012-2013	[145]
Human	class 1 integron	*aadA7*	Algeria	2012-2013	[146]
Human	class 1 integron	*bla*$_{IMP-87}$, *ant(2")-Ia*, *bla*$_{OXA-10}$, *aac(6')-Ib3*	China	2014-2015	[147]
Human	class 1 integron	*aacA29b*, *bla*$_{VIM-2}$, *aacA29a*, *qacEΔ1*, *sul1*	Netherland	2015	[148]
Human	class 1 integron	*aacA29e*, *bla*$_{VIM-2}$, *aacA29e*, *qacEΔ1*, *sul1*	Netherland	2015	[148]
Human	class 1 integron	*aacA4'-8*, *bla*$_{CARB-2}$, *aadA2*, *qacEΔ1*, *sul1*	Netherland	2015	[148]
Human	class 1 integron	*AmpC*	Korea	2011-2014	[149]
Human	class 1 integron	*bla*$_{VIM-2}$, *qacEΔ1*, *sul1*	India	2013-2014	[150]

Table S2. Characterization of mobile genetic elements (MGEs), plasmids, associated with antibiotic resistance genes (ARGs) in *P. aeruginosa*

Source	MGEs	ARGs associated	Geographic region	Year	Reference
Human	plasmid	*aph-(6)-ld, aph-(3'')-lb, aac-(3'')-lld, qnrS1, bla*$_{VIM-2}$	Argentina	2012	[151]
Human	plasmid	*bla*$_{IMP-10}$, *aacA7*	China	2012	[55]
Human	plasmid	*bla*$_{GES-5}$, *bla*$_{GES-5}$, *bla*$_{GES-5}$, *bla*$_{GES-5}$	China	2012	[60]
Human	plasmid	*bla*$_{KPC-2}$	Argentina	2008, 2018	[152]
Human	plasmid	*aph(6)-Id*, *aph(3'')-Ib*, *sul1*, *floR*, *tetR*, *tet*(G), *ant(4')-IIb*, *sul1*, *emrE*, *aadA*, *bla*$_{OXA-10}$, *cmlA5*, *arr2*, *ant*(2')-1a, *bla*$_{VEB-1}$	Thailand		[70]
Human	plasmid	*aadA1*, *qnrS2*, *mph*(A), *cmlA1*, *tet*(G), *sul1*, *aph(3')-IIb*, *bla*$_{OXA-396}$, *aac*(3)-IId, *bla*$_{PAO}$, *crpP*, *fosA*, *catB7*, *bla*$_{KPC-2}$	China	2010, 2021	[153]
		*aac(6')-II*a, *ant(2'')-Ia*, *aph(3')-IIb*, *aph(3')-VI*, *sul1*, *bla*$_{CARB-2}$, *crpP*, *bla*$_{PAO}$, *bla*$_{OXA-486}$, *fosA*, *catB7*, *bla*$_{KPC}$			
Human	plasmid	*bla*$_{GES}$, *crpP*, *bla*$_{KPC}$	China		[154]
Human	plasmid	*qacEΔ1*, *aadB*, *aadA10e*, *sul1*, *bla*$_{OXA10}$	U.S.A	1999, 2002, 2003, 2009, 2013, 2015, 2016, 2018	[77]
Human	plasmid	*bla*$_{VEB-1-like}$, *bla*$_{OXA-10-like}$	Thailand	1994, 1996	[155]
Human	plasmid	*bla*$_{KPC}$, *sul1*, *amlA1*, *aadB*	China		[156]
	plasmid	*sul1*, *smlA1*, *aadB*,			
Human	plasmid	*bla*$_{KPC}$	Colombia	2006, 2007, 201	[157]
Human	plasmid	*bla*$_{VIM}$, *aacA7*, *aacC1*, *aacA4*, *qacEΔ1*, *sul1*	Portugal	2008, 2010	[79]
Human	plasmid	*bla*$_{KPC}$	Europe		[158]
Human	plasmid	*bla*IMP, *bla*$_{CTX}$, *aacC2*, *rmtB*	China	2016	[80]
Human	plasmid	*bla*$_{NDM}$, bla$_{VIM}$, *qnr*, *aad*	Nigeria	2018, 2019	[159]
Human	plasmid	*bla*$_{SHV-1}$, *bla*$_{TEM-1}$, *bla*$_{OXA-10}$, *bla*$_{PSE-1}$	Iran	2017, 2018	[160]
Human	plasmid	*bla*$_{OXA-935}$	U.S.A	1999, 2002, 2003, 2009, 2013, 2015, 2016, 2018	[161]

Environment	plasmid	*aph*, *sul1*, *qacEΔ1*, *dfrB2*, *bla*$_{OXA-10}$			[162]
Chicken	plasmid	*aacA4*, *bla*$_{IMP-45,}$ *bla*$_{OXA-1}$, *catB3*, *sul1*, *armA*, *msr*(E), *mph*(E), *aph*(3')-1c, *tet*(C), *aac*(3)-IVa, *aph*(4)-Ia, *tet*(X6), *floR*, *strB*, *aph*(3')-Via, *strB*, *strA*, *sul1*, *dfrA22e*, *arr-2*, *qnrVC1*, *tmexC3*, *tmexD3*, *toprJ3*	China	2019	[163]
	plasmid	*strB*, *strA*, *aac*(3)-IVa, *aph*(4)-Ia, *tet*(X6), *floR*, *strB*, *aph*(3')-VI, *strB*, *strA*			
Human	plasmid	*bla*$_{KPC-2}$	China	2021	[164]
Human	plasmid	*aacA4*	China		[165]
Human	plasmid	*acrB*, *sul1*, *qacEΔ1*, *cmlA1*, *dfrA15*, *strB*, *strA*, *bla*$_{NPS-1}$	India	1997	[166]
Human	plasmid	*bla*$_{IMP-1}$	Mexico	2013-2015	[97]
Human	plasmid	*bla*$_{PAU-1}$	China	2009-2012	[167]
	plasmid	*sul1*, *qacEΔ1*, *aadA1*, *aphA15*, *aacA4*, *bla*$_{VIM-1}$, *strA*, *strB*			
Human	plasmid	*bla*$_{KPC-2}$	China	2019	[168]
Human	plasmid	*aac(6')-Ib-7*, *bla*$_{IMP-45}$, *bla*$_{OXA-1}$, *catB3*, *qacEΔ1*, *sul1*, *armA*, *msrE*, *mphE*, *sul1*, *qacEΔ1*, *dfrA22*, *arr-2*, *qnrVC1*,	China	2015-2017	[101]
Human	plasmid	*bla*$_{KPC-2}$	Mexico	2023	[46]
	plasmid	*bla*$_{OXA-101}$, *bla*$_{CTX-M-30,}$ *bla*$_{TEM-1b}$			
Human	plasmid	*bla*$_{IMP-56}$, *aadA1*, *bla*$_{OXA-2}$	Mexico	2023	[102]
	plasmid	*AmpC*			
Human	plasmid	*bla*$_{OXA-10}$, *aac(6')-Ib*, *bla*$_{IMP-8}$, *aac(6')-Ib*, *aph*(3')-XV, *aadA10*, *bla*$_{OXA-2}$, *sul1*	Germany	2009	[104]
Human	plasmid	*bla*$_{CTX-M}$, *bla*$_{NDM}$, *bla*$_{KPC}$	Finland	2018	[169]
Environment	plasmid	*APH(3')-IIa*			[170]
Human	plasmid	*bla*$_{SIM-2}$, *gcu104*, *ereA1*, *catB3q*, *gcu161*, *arr3*, *aadA1a*, *qacEΔ1*, *sul1*	China	2012	[171]
Human	plasmid	*bla*$_{KPC-113}$	China	2020	[172]
Human	plasmid	*bla*$_{KPC}$, *aph*(3')-Iib, *aph*(3')-Vi, *fosA*, *catB*	Brazil	2014	[173]
Human	plasmid	*bla*$_{KPC-2}$	Brazil	2018	[174]
Human	plasmid	*bla*$_{KPC-2}$	China	2018	[175]
Human	plasmid	*bla*$_{KPC-2}$	Brazil	2020	[176]
Human	plasmid	*bla*$_{IMP-4}$	China	2009-2013	[177]
Human	plasmid	*aadB*, *bla*$_{VIM-1}$	Spain	2006-2007	[120]
		bla$_{VIM-1}$, *aacA4*, *bla*$_{VIM-1}$, *bla*$_{VIM-1}$, *aadA1*, *qacEΔ1*, *sul1*			
Human	plasmid	*aacA7*, *bla*$_{VIM-2}$, *aacC1*, *aacA4*, *qacEΔ1*, *sul1*	Portugal	1995-2014	[178]
Human	plasmid	*aacA4*, *bla*$_{IMP-5}$, *aacA4*	China	2000	[128]
Human	plasmid	*bla*$_{OXA-198}$	Belgium	2010-2013	[134]
Human	plasmid	*bla*$_{GES-2}$	South Africa	2000	[179]

Human	plasmid	bla$_{KPC-2}$	China	2009	[180]
Human	plasmid	*qnrVC1*, *dfrA47*, *sul1*, *mph*(E), *armA*, *sul1*, *aadA25*, *cmlA1*, *aadB*, *aac(6')-II*, *bla*$_{AFM-2}$, *sul1*, *dfrA27*, *arr-3*, *bla*$_{OXA-246}$, *cmlAB*, *aacA4*	China	2021	[181]
Human	plasmid	*bla*$_{IMP-45}$, *aac(6')-Ib3*, *bla*$_{OXA-1}$, *catB3*, *qnrVC6*, *armA*, *msr*(E), *mph*(E), *aph*(3')-Ia, *tetC*, *tetR*, *aac(6')-Ib3*, *floR*, *mexC-mexD-oprJ*, *fosA*	China	2014-2015	[147]
Human	plasmid	*bla*$_{KPC-2}$, *bla*$_{KPC-2}$<br>*bla*$_{KPC-33}$	Chile	2019	[182]
Human	plasmid	*bla*$_{TEM-1}$, *bla*$_{KPC-1}$	China	2010	[183]
Human	plasmid	*bla*$_{KPC-2}$	Brazil	2011	[184]
Human	plasmid	*bla*$_{KPC-2}$	China	2015	[185]
Human	plasmid	*bla*$_{NDM-1}$	India	2011-2012	[186]
Human	plasmid	*aac(6')-I, aac(6')- II*	India	2003	[187]

Table S3. Characterization of mobile genetic elements (MGEs), transposons, associated with antibiotic resistance genes (ARGs) in *P. aeruginosa*

Source	MGEs	ARGs associated	Geographic region	Year	Reference
Human	Tn*4371*	*bla*$_{SPM-I}$	Brazil	2017	[52]
Human	Tn*7339*	*bla*$_{IMP-I0,}$ *aacA7*	China	2012	[55]
Human	Tn*6584*	*bla*$_{GES-I}$, *acc*(6')-*Ib*, *aph*(3')-*XV*	China	2010	[57]
Human	Tn*3*	*qnrVCI*, *aph*(6')-*Id*, *tetG*	Thailand	1997, 2018	[188]
	Tn*3*	*bla*$_{TEM-IB}$			
	Tn*3*	*bla*$_{OXA-I0}$			
Human	Tn*402*-like, Tn*21*	*bla*$_{VIM-2}$			[189]
Human	Tn*6609*	*aadA7*, *qacEΔ1*, *sulI*	Cyprus		[63]
Human	Tn*4401b*	*bla*$_{KPC-2}$	Argentina	2008, 2018	[152]
Human	Tn*3*	*aph(6)-Id*, *qnrVCI*, *tet*(G), *tet*(R)	India, Australia	1992, 2018	[190]
Human	Tn*5393*	*aph(6)-Id*, *aph(3'')-Ib*	Thailand		[70]
	Tn*As3*	*sulI*			
Human	Tn*3*like	*bla*$_{KPC-2}$	China	2010, 2021	[153]
Human	Tn*3*, Tn*1403*	*bla*$_{KPC-2}$	China		[156]
Human	Tn*4401b*	*bla*$_{KPC-2}$	Colombia	2006, 2007, 2011	[157]
Human	Tn*6356*	*bla*$_{VIM-2}$, *aacA7*, *aacC1*, *aacA4*, *qacEΔ1*, *sul1*	Portugal	2008, 2010	[79]
Human	Tn*6394*	*bla*$_{IMP-1}$, *bla*$_{CTX-M-14}$, *aacC2*, *rmtB*	China	2016	[80]
Human	Tn*402*	*bla*$_{VIM-2}$, *AmpD*	Spain	2020	[191]
	Tn*402*	*bla*$_{IMP-13}$			
	Tn*402*	*bla*$_{IMP-28}$			
Human	Tn*501*-like	*aacA7*, *bla*$_{VIM-2}$, *dfrB5*, *aacC-A5*, *qacEΔ1*, *sul1*, *cmlA9*, *qacEΔ1*, *tetR*, *tetA*(G), *sul1*	U.S.A	2007	[89]
Environment	Tn*402*-like	*aph*, *sul1*, *qacEΔ1*, *dfrB2*, *bla*$_{OXA-10}$			[162]
Chicken	Tn*6485b*	*aac*$_{A4}$, *bla*$_{IMP-45}$, *bla*$_{OXA-1}$, *catB3*, *sul1*, *armA*, *msr*(E), *mph*(E), *aph*(3')-*Ic*	China	2019	[163]
Human, Environment	Tn*4371*	*bla*$_{NDM-1}$, *msr*(E), *floR*	Singapore	2019, 2020	[91]
Environment	Tn*3*	*vanRA*, *van*SA, *van*HA, *van*A, *vanXA*, *van*YA	India	2019, 2020	[192]
Human	Tn*3*	*acrB*, *sul1*, *qacEΔ1*, *cmlA1*, *dfrA15*, *strB*, *strA*, *bla*$_{NPS-1}$	India	1997	[166]
Human	Tn*3*	*bla*$_{PAU-1}$	China	2009-2012	[167]
Human	Tn*6532*, Tn*6809*, Tn*6346*	*aadB*, *qacEΔ1*, *sul1*	China	2011-2019	[193]
Human	Tn*6203*	*bla*$_{KPC-2}$	China	2019	[168]
Human	Tn*7517*	*bla*$_{TEM-1b}$, *aph*(3')-Vi, *bla*$_{VIM-2}$, *qacEΔ1*, sul1, *bla*$_{PER-1}$, *qacEΔ1*, sul1	Uruguay	2016, 2021	[194]
Human	transposon	*bla*$_{OXA-101}$, *bla*$_{CTX-M-30}$, *bla*$_{TEM-1b}$	Mexico	2023	[46]
Human	Tn*3*	*bla*$_{KPC-2}$	China	2018	[175]
Human	Tn *6786*	*crpP*	China	2010-2019	[195]

Human	Tn*6346*-like	*bla*$_{AFM-1}$, *floR*	China	2017	[196]
Human	Tn*2345*	*bla*$_{PER-1}$	France	2001	[123]
Human	Tn*1721*-like	*bla*$_{PAC-1}$, *qacEΔ1*, *sul1*	Mauritius, Afghanistan	2017-2019	[197]
Human	Tn*1403*-related	*aacA4*, *bla*$_{OXA-677}$, *aadA1*	China	2018	[198]
	Tn*1403*-related	*aacA4*, *bla*$_{OXA-101}$, *aadA5*			
	Tn*1403*-related	*bla*$_{OXA-246}$, *aacA3, aadA13*			
Human	Tn*4401b*	*bla*$_{KPC-2}$, *bla*$_{KPC-2}$	Chile	2019	[182]
	Tn*4401b*	*bla*$_{KPC-33}$			
Human	Tn*5051*-like	*bla*$_{CARB-2}$, *aadA2*, *qacEΔ1*, *sul1*	Netherland	2015	[148]

Table S4. Characterization of mobile genetic elements (MGEs), insertion sequences (ISs), associated with antibiotic resistance genes (ARGs) in *P. aeruginosa*

Source	ISs	ARGs associated	Geographic region	Year	Reference
Human	IS*Kpn19*, IS*26*	*qnrS1*	Argentina	2012	[151]
Human	IS*1326* , IS*1353*	*aadA1, qacEΔ1, sul1*	Russia	2013-2016	[139]
	IS*1326*, IS*1353*	*bla*$_{IMP-1}$, *aac(6')-Iae, aadA1, qacEΔ1, sul1*			
Human	IS*CR14*	*rmtD*	Brazil	2017	[52]
Human	IS*Psy6*, IS*Pa21*	*bla*$_{GES-1}$, *acc*(6')-*Ib*, *aph*(3')-*XV*	China	2010	[57]
Human	IS*Aba125*, IS*91*	*bla*$_{NDM-1}$			[189]
Human	IS*Pa51* , IS*Pa52*	*oprD*	Poland	2009, 2010	[199]
Human	insertion sequence	*aph(3')-IIb, bla*$_{OXA-50}$	India, Australia		[200]
Human	ISPa11	*mexZ, mexR, mexT, ampR, pmrA*	Canada	1997	[201]
Human	IS*6100*	*bla*$_{KPC}$	China		[156]
Human	insertion sequence	*oprD*			[202]
Human	IS*6100*	*bla*$_{VIM-2}$, *AmpD*	Spain	2020	[191]
	IS*Pa17*	*bla*$_{IMP-13}$			
Human	IS*6100*, IS*Kpn27*, IS*Kpn6*	*bla*$_{KPC}$	China	2021	[164]
Human	IS*Pa8*	*oprD*	U.S.A		[203]
Human	IS*Kpn6*-like, IS*kpn8*-like	*bla*$_{KPC-2}$	China	2009-2018	[24]
Human	insertion sequence	*oprD*	U.S.A		[37]
Human	IS*Pa46*	*oprD*	France	2011	[204]
Human	IS*RP10*	*oprD*	China	2009-2010	[205]
Human	IS*Pa1328*, IS*Psme1*, IS*Pa26*, IS*Pst2*, IS*Pa195*	*oprD*	Russia	2012-2017	[113]
Human	IS*Pa45*	*oprD*	Spain	2008-2010	[114]
Human	IS*Pa195*	*oprD*	Russia	2013	[206]
Human	IS*26*, IS*Kpn27*, IS*Kpn6*, IS*26*, IS*26*	*bla*$_{KPC-2}$	China	2018	[175]
Human	IS*Pa1328*	*oprD*	France		[207]
Human	IS*Ppu21*	*oprD*	Iran	2014-2015	[208]
Human	IS*21*	*mexR*	France	1996	[209]
Human	IS*PA26*	*oprD*	South Africa	2000, 2004, 2006	[210]
	IS*Pa133*	*oprD*			[211]
Human	IS*1411*	*oprD*	China	2011-2016	[212]

Table S8. Mobile ARGs, their major ST carriers, MGEs responsible for their dissemination and their One Health origin based on genome database survey and systematic literature survey

Antibiotic class	ARG name	Genome database survey							Systematic literature analysis	
		Proportion in all genomes (%)	No. of types of STs carrying the ARG	Major STs carriers of the ARG, with more than 10 genomes (proportion of the ARG-carrying genomes per all genomes of each ST)	Proportion in One-health sector (%)			Associated MGEs	ARGs linked to MGES	Associated MGEs
					Human	Animal	Environment			
Amino-glycoside	*aac(6')-Ib*	14.7	93	235 (6.52), 111 (30.66), 621 (18.85), 244 (8.33), 277 (14.38), 308 (116.41), 357 (15.79), 316 (5.42), 348 (36.08), 664 (26.72), 175 (16), 233 (16.67), 253 (34.94), 773 (18.58), 179 (10.53), 654 (77.39), 17 (23.64), 309 (63.16), 446 (27.69)	16.1	5.2	11.5	integron, transposon, plasmid	Yes	integron, plasmid, transposon
	aph(6)-Id	6.7	99	111 (2.68), 179 (9.52), 233 (30.14), 235 (12.11), 244 (13.91), 308 (32.82), 313 (16.39), 316 (11.49), 357 (28.42), 446 (11.58), 463 (4.81), 644 (84.62), 654 (92.73), 823 (77.78), 1047 (111.11), 1203 (77.78), 1418 (94.12)	7.1	12.6	4.3	integron, transposon, plasmid		
	aph(3'')-Ib	6.3	92	179 (8.93), 233 (26.03), 235 (10.55), 244 (15.41), 308 (32.82), 316 (10.34), 357 (26.78), 463 (4.81), 654 (90.91), 823 (77.78), 1047 (111.11), 1203 (83.33), 1418 (94.12)	6.8	8.7	4.3	integron, transposon, plasmid		
	ant(3'')-IIa	5.4	71	175 (87.7), 233 (15.1), 235 (17.4), 244 (3.8), 298 (17.3), 357 (41.5), 395 (7.2), 463 (5.8)	7.3	4.3	6.2	integron, transposon, plasmid	Yes	integron, plasmid, transposon, IS
	ant(2'')-Ia	6.4	71	111 (2.4), 167 (72.2), 179 (7.1), 233 (16.4), 235 (19.7), 244 (4.5), 309 (33.3), 357 (50.8), 485 (45.5), 621 (78.3), 1976 (86.7), 2731 (200)	6.0	1.3	2.7	integron, transposon, plasmid	Yes	integron, plasmid, transposon
	aadA2	2.6	27	111 (20.19), 233 (62.33), 235 (3.52), 412 (55.17)	2.9	3.9	0.3	integron, transposon	Yes	integron, transposon
	aac(6')-Il	2.7	23	233 (52.05), 235 (13.87), 308 (13.74), 357 (24.04)	3.4	0.9	1.4	integron, transposon	Yes	integron, plasmid, transposon
	aph(3')-VIa	2.1	42	235 (7.03), 357 (17.49), 654 (54.55)	2.6	3.0	0.0	integron, transposon, plasmid		
	aac(3)-Id	1.4	7	233 (58.9), 235 (2.34), 308 (11.45), 823 (61.11)	1.8	0.9	0.8	integron		
	aph(3')-Ia	1.3	31	235 (2.73), 485 (45.45), 1971 (136.36)	1.3	3.5	1.9	plasmid		
	aac(3)-IIa	1.0	28	235 (4.1), 1418 (94.12)	1.3	0.4	0.2	transposon, plasmid	Yes	plasmid, transposon
	rmtB	0.9	21	773 (61.54), 1418 (94.12)	1.2	0.0	0.2	transposon		
	ant(4')-IIb	0.8	23	233 (7.53), 654 (23.64), 664 (60.53)	0.9	0.0	2.4	transposon		
	aac(6')-IIa	0.8	20	235 (3.13), 463 (10.58), 1418 (88.24)	1.0	0.9	0.0	transposon, plasmid		
	aac(6')-Ib7	0.6	13	111 (5.84), 357 (5.46)	0.6	0.0	0.5	integron, transposon		
	aac(6')-29a	0.5	3	111 (11.68)	0.5	0.0	0.3	integron, transposon		
	aadA13	0.6	13	175 (13.11), 1418 (94.12)	0.8	0.0	0.0	transposon	Yes	integron, transposon
	aac(6')-31	0.4	2	235 (4.49)	0.4	0.0	0.0	integron		
β-Lactam	*bla*$_{VIM-1}$	5.8	55	111 (41.12), 175 (31.97), 179 (5.95), 233 (71.92), 235 (8.98), 308 (7.63), 357 (5.46), 654 (43.64), 823 (105.56)	5.8	1.3	3.0	integron, transposon, plasmid	Yes	integron, plasmid
	bla$_{OXA-10}$	5.4	61	111 (3.89), 175 (9.02), 233 (14.38), 235 (11.52), 244 (11.65), 260 (19.05), 277 (51.55), 298 (19.75), 308 (7.63), 357 (49.18), 664 (71.05), 1418 (94.12), 1976 (86.67)	6.5	0.4	3.8	integron, transposon, plasmid	Yes	integron, plasmid, transposon
	bla$_{KPC-2}$	3.8	45	235 (3.52), 244 (6.39), 463 (98.56), 485 (45.45), 1076 (31.58), 1212 (67.86)	5.2	0.0	0.0	plasmid	Yes	plasmid, transposon, IS
	bla$_{OXA-2}$	3.3	38	111 (3.65), 235 (12.89), 309 (17.33), 357 (9.29), 446 (10.53), 621 (78.26)	4.0	0.9	0.0	integron, transposon	Yes	integron, plasmid
	bla$_{GES-1}$	2.7	31	235 (28.13), 309 (32), 654 (25.45), 1203 (55.56)	2.8	0.4	1.9	transposon	Yes	integron, plasmid, transposon, IS
	bla$_{ERP-1}$	2.4	45	155 (25.25), 316 (6.32), 357 (7.1), 386 (70.59), 395 (7.23), 560 (41.94)	2.3	2.6	3.3	integron		
	bla$_{OXA-1}$	2.1	39	233 (68.49), 235 (2.93), 664 (57.89)	2.6	2.6	0.8	integron, transposon, plasmid	Yes	integron, plasmid, transposon
	bla$_{CARB-1}$	1.9	23	111 (23.11), 235 (2.93), 463 (11.54)	2.1	0.4	0.0	integron, transposon		
	bla$_{NDM-1}$	1.5	13	308 (14.5), 357 (19.67), 644 (92.31), 654 (32.73), 773 (63.08)	1.9	0.0	1.1	integron, transposon	Yes	integron, plasmid, transposon, IS
	bla$_{VEB-1}$	1.4	11	235 (4.88), 357 (45.9)	1.7	0.0	1.3	integron, transposon, plasmid	Yes	integron, plasmid

	$bla_{\text{IMP-1}}$	1.3	20	111 (9.73), 235 (5.08), 357 (8.2), 1976 (80)	1.6	0.0	0.0	plasmid	Yes	integron, plasmid, transposon
	$bla_{\text{PER-1}}$	0.9	23	235 (3.32), 244 (5.26), 1418 (94.12)	1.1	0.0	0.0	transposon, plasmid		
	$bla_{\text{OXA-9}}$	0.5	3	111 (12.41)	0.6	0.0	0.0	integron, transposon		
	$bla_{\text{IMP-9}}$	0.3	20		0.4	1.7	0.0	plasmid	Yes	integron, plasmid, transposon
	$bla_{\text{AFM-1}}$	0.2	6	463 (7.69)	0.3	0.0	0.0	plasmid		
MLSB	*msrE*	1.1	31	308 (15.27), 463 (7.69)	1.2	1.7	1.3	transposon, plasmid		
	mphE	0.8	31	463 (7.69)	1.0	1.7	0.3	transposon, plasmid	Yes	plasmid, transposon
Sulfonamide	*sul1*	39.2	150	17 (10.33), 111 (83.21), 155 (7.92), 167 (61.11), 175 (210.66), 179 (19.05), 233 (202.05), 234 (55.88), 235 (144.73), 244 (29.7), 253 (10.54), 260 (50.79), 277 (125.77), 282 (40.63), 292 (137.5), 298 (25.93), 308 (38.93), 309 (46.67), 313 (16.39), 316 (164.37), 348 (38.55), 357 (131.15), 360 (50), 395 (25.3), 412 (55.17), 446 (43.16), 463 (29.33), 485 (45.45), 621 (96.52), 644 (230.77), 654 (160), 664 (100), 697 (120), 708 (70.59), 773 (120), 1047 (166.67), 1203 (161.11), 1418 (123.53), 1971 (136.36), 1976 (100), 2592 (150), 3986 (333.33)	44.6	16.5	17.9	integron, transposon, plasmid	Yes	integron, plasmid, transposon, IS
Diamino-pyrimidine	*dfrB5*	1.5	12	233 (60.27), 235 (2.34), 308 (11.45)	1.9	0.9	0.8	integron		
	dfrA5	0.7	15	233 (8.9), 235 (3.13), 644 (7.69), 883 (16.67)	0.7	0.0	1.7	transposon		
	dfrB2	0.6	7	357 (27.32)	0.7	0.0	1.3	integron		
	dfrA10	0.3	9	233 (7.53)	0.2	0.0	1.6	transposon		
	dfrA27	0.2	9		0.3	0.0	0.0	plasmid		
Phenicol	*floR*	6.4	52	233 (87.67), 235 (21.68), 308 (24.43), 316 (48.85), 357 (14.75), 644 (115.38), 654 (54.55), 664 (60.53), 773 (63.08), 1203 (105.56)	7.4	5.7	5.5	integron, transposon, plasmid	Yes	integron, plasmid, transposon
	cmlA1	3.8	45	233 (63.7), 235 (15.63), 357 (13.66), 463 (7.21), 1418 (94.12), 1971 (127.27)	4.7	3.0	0.5	integron, transposon, plasmid	Yes	integron, plasmid, transposon
	catB3	1.2	34	235 (7.23), 485 (45.45)	1.5	1.7	0.0	plasmid	Yes	integron, plasmid, transposon
	cmlB	0.5	1	111 (12.17)	0.6	0.0	0.0	integron, transposon		
Quinolone	*qnrVC1*	3.5	46	233 (11.64), 308 (19.85), 316 (68.39), 357 (8.2), 773 (73.85), 1203 (55.56)	4.4	3.0	3.0	integron, transposon, plasmid	Yes	integron, plasmid, transposon
MDR	*qacEΔ1*	17.0	104	111 (55.47), 175 (84.43), 179 (6.55), 233 (58.9), 235 (78.91), 244 (11.65), 253 (7.53), 260 (20.63), 277 (25.77), 298 (22.22), 308 (13.74), 316 (13.79), 348 (28.92), 357 (51.37), 395 (9.64), 412 (55.17), 446 (18.95), 463 (5.29), 485 (42.42), 621 (81.74), 773 (60), 1203 (77.78), 1418 (82.35)	19.0	5.2	9.8	integron, transposon, plasmid	Yes	integron, plasmid, transposon, IS
	qacE	1.5	28	111 (3.89), 175 (22.95), 235 (10.94)	1.1	0.0	0.9	integron, transposon	Yes	integron, plasmid
etc	*brp*	0.7	10	357 (16.94), 463 (7.69)	0.9	0.0	0.0	transposon, plasmid		
